# Altered substrate metabolism in neurodegenerative disease: new insights from metabolic imaging

**DOI:** 10.1186/s12974-021-02305-w

**Published:** 2021-10-28

**Authors:** Nicholas R. W. Cleland, Saif I. Al-Juboori, Evgenia Dobrinskikh, Kimberley D. Bruce

**Affiliations:** 1 Endocrinology, Metabolism and Diabetes, Division of Endocrinology, Metabolism and Diabetes, Department of Medicine, University of Colorado Anschutz Medical Campus, Aurora, USA; 2 Section of Neonatology, Department of Pediatrics, University of Colorado Anschutz Medical Campus, Aurora, USA; 3 Division of Pulmonary Sciences and Critical Care, Department of Medicine, University of Colorado Anschutz Medical Campus, Aurora, USA; 4 School of Medicine, University of Colorado Anschutz Medical Campus, Aurora, USA

**Keywords:** Neurodegenerative disease, Metabolic imaging, Brain metabolism, Neurons, Glia

## Abstract

Neurodegenerative diseases (NDs), such as Alzheimer’s disease (AD), Parkinson’s disease (PD) and multiple sclerosis (MS), are relatively common and devastating neurological disorders. For example, there are 6 million individuals living with AD in the United States, a number that is projected to grow to 14 million by the year 2030. Importantly, AD, PD and MS are all characterized by the lack of a true disease-modifying therapy that is able to reverse or halt disease progression. In addition, the existing standard of care for most NDs only addresses the symptoms of the disease. Therefore, alternative strategies that target mechanisms underlying the neuropathogenesis of disease are much needed. Recent studies have indicated that metabolic alterations in neurons and glia are commonly observed in AD, PD and MS and lead to changes in cell function that can either precede or protect against disease onset and progression. Specifically, single-cell RNAseq studies have shown that AD progression is tightly linked to the metabolic phenotype of microglia, the key immune effector cells of the brain. However, these analyses involve removing cells from their native environment and performing measurements in vitro, influencing metabolic status. Therefore, technical approaches that can accurately assess cell-specific metabolism in situ have the potential to be transformative to our understanding of the mechanisms driving AD. Here, we review our current understanding of metabolism in both neurons and glia during homeostasis and disease. We also evaluate recent advances in metabolic imaging, and discuss how emerging modalities, such as fluorescence lifetime imaging microscopy (FLIM) have the potential to determine how metabolic perturbations may drive the progression of NDs. Finally, we propose that the temporal, regional, and cell-specific characterization of brain metabolism afforded by FLIM will be a critical first step in the rational design of metabolism-focused interventions that delay or even prevent NDs.

## Background

Despite being only 2% of the body’s mass, the brain consumes 20% of the body’s oxygen intake and 25% of its glucose intake [1]. This energy utilization is not uniform, with 70–80% of total energy consumed via neurons, and microglia, astrocytes and oligodendrocytes using the remaining portion [2–4]. Although the brain primarily consumes glucose to meet its energy demands, in disease states or starvation energy utilization can shift to other substrates [5–9]. During aging, glucose metabolism in the brain is impaired, a phenomenon that is exacerbated in neurodegenerative (ND) and neuroinflammatory diseases (NID) such as Alzheimer’s disease (AD), Parkinson’s disease (PD) and multiple sclerosis (MS). Specifically in AD, several changes to overall brain metabolism have been observed, including dysregulated oxidative metabolism, amino acid metabolism and creatine degradation [10], and impaired glucose transport [11]. In particular, the pathogenesis of AD is complex, and despite major research efforts, successful therapeutics that can prevent, or delay AD are lacking. Since perturbed brain metabolism has been repeatedly implicated in AD, understanding changes in substrate utilization prior to and during the development and progression of AD may highlight critical mechanisms that can be exploited to diagnose or treat AD.

Despite changes in brain metabolism being a potential mechanism driving the development of NDs, the field has been stymied by the lack of techniques that can accurately measure brain metabolism with sufficient temporal, spatial, and cell-specific resolution. On one hand, it is becoming widely appreciated that both neurons and glial cells, which have very different metabolic needs, contribute to disease pathology. Recent methodological advances have facilitated the identification of microglial subpopulations with defined metabolic phenotypes that may protect against or precede AD [12, 13]. However, these measurements have been made in isolated cells that have been subjected to mechanical or enzymatic stress, which likely modifies their metabolic state, underscoring the need for methodologies that can determine cell-specific metabolic changes in situ.

Specific brain regions have different cellular composition, functions, and therefore metabolic needs. Hence metabolic perturbations in certain regions may have a greater impact on disease state and severity. Nonetheless, existing “metabolic imaging” modalities, such as magnetic resonance imaging (MRI), magnetic resonance spectroscopy (MRS), and positron emission tomography (PET), have provided important insights regarding regional changes in metabolic processing that occur preceding and during NDs, such as AD [14]. Although these techniques are becoming increasingly advanced, they cannot discriminate between regional and cell-specific metabolic aberrations. In contrast, the development of two-photon imaging and fluorescence lifetime imaging microscopy (FLIM), enables direct measurement of the endogenous metabolic fluorophores, such as reduced nicotinamide adenine dinucleotide (NADH) and flavin adenine dinucleotide (FAD), which have very distinct lifetimes in free and bound-to-enzyme forms [15]. The ratio of free and bound-to-enzyme NADH or FAD can be determined within an individual cell or on a cell cluster in tissue, and therefore has the potential to measure cell and region-specific changes in brain metabolism that may precede or drive AD onset and progression. Here, we revisit our understanding of brain metabolism and review recent findings suggesting that changes in brain metabolism may be a potential target for the treatment of ND. We also highlight recent advances in metabolic imaging that enable precise monitoring of brain metabolism, guiding the design of interventions that may prevent or improve outcomes for individuals with NDs such as AD.

## Metabolism in the homeostatic brain

### Overview of metabolic processes in the brain

Energy usage is dependent on a given cell’s microenvironment, the substrates available for metabolism, and the role the cell has in maintaining homeostasis or fighting off disease. Generally, the pathways available for energy production are glycolysis, the tricarboxylic acid cycle (TCA cycle), and oxidative phosphorylation (OXPHOS). Glycolysis takes 1 glucose molecule, 2 nicotinamide adenine dinucleotide (NAD^+^) molecules, 2 adenine diphosphate (ADP), and 2 phosphate groups to yield 2 pyruvate molecules, 2 reduced nicotinamide adenine dinucleotide (NADH) molecules, 2 water (H_2_O) molecules, and 4 adenine triphosphate (ATP) molecules. These pyruvate molecules are converted to two acetyl coenzyme A (acetyl-CoA) before entering TCA cycle, where they undergo a series of reactions yielding either 2 guanosine triphosphate (GTP) or ATP molecules, 6 NADH molecules, 2 ubiquinone (QH_2_) molecules, 2 FADH_2_ molecules, and 4 carbon dioxide (CO_2_) molecules. The reducing agents generated by these pathways are used in oxidative phosphorylation to break covalent bonds in molecular oxygen (O_2_), converting chemical energy into a proton gradient. ATP synthase, an enzyme found in the mitochondrial inner membrane, then uses the proton gradient to phosphorylate ADP to ATP, a form of chemical energy that can be used throughout the cell.

Another pathway important in homeostasis and cell repair is the pentose phosphate pathway (PPP). This pathway is a parallel of glycolysis and converts glucose-6-phosphate to pentoses, and ribulose-5-phosphate which can be used in the synthesis of nucleotides. The PPP can be broken up into an oxidative phase where 2 nicotinamide adenine dinucleotide phosphate (NADP^+^) are reduced to NADPH via the oxidation of glucose-6-phosphate, and a non-oxidative phase where pentoses are generated. Unlike glycolysis, the function of the PPP is anabolic rather than catabolic.

The brain is composed of neurons and glial cells. Glial cells can be further categorized as ependymal cells, oligodendrocytes, microglia, and astrocytes. Below we will examine the preferred substrate utilization within neurons, astrocytes, microglia, and oligodendrocytes and metabolic cross-talk between them. We will also evaluate how alterations in these processes may contribute to ND.

### Astrocytes and neurons

**Fig. 1:**
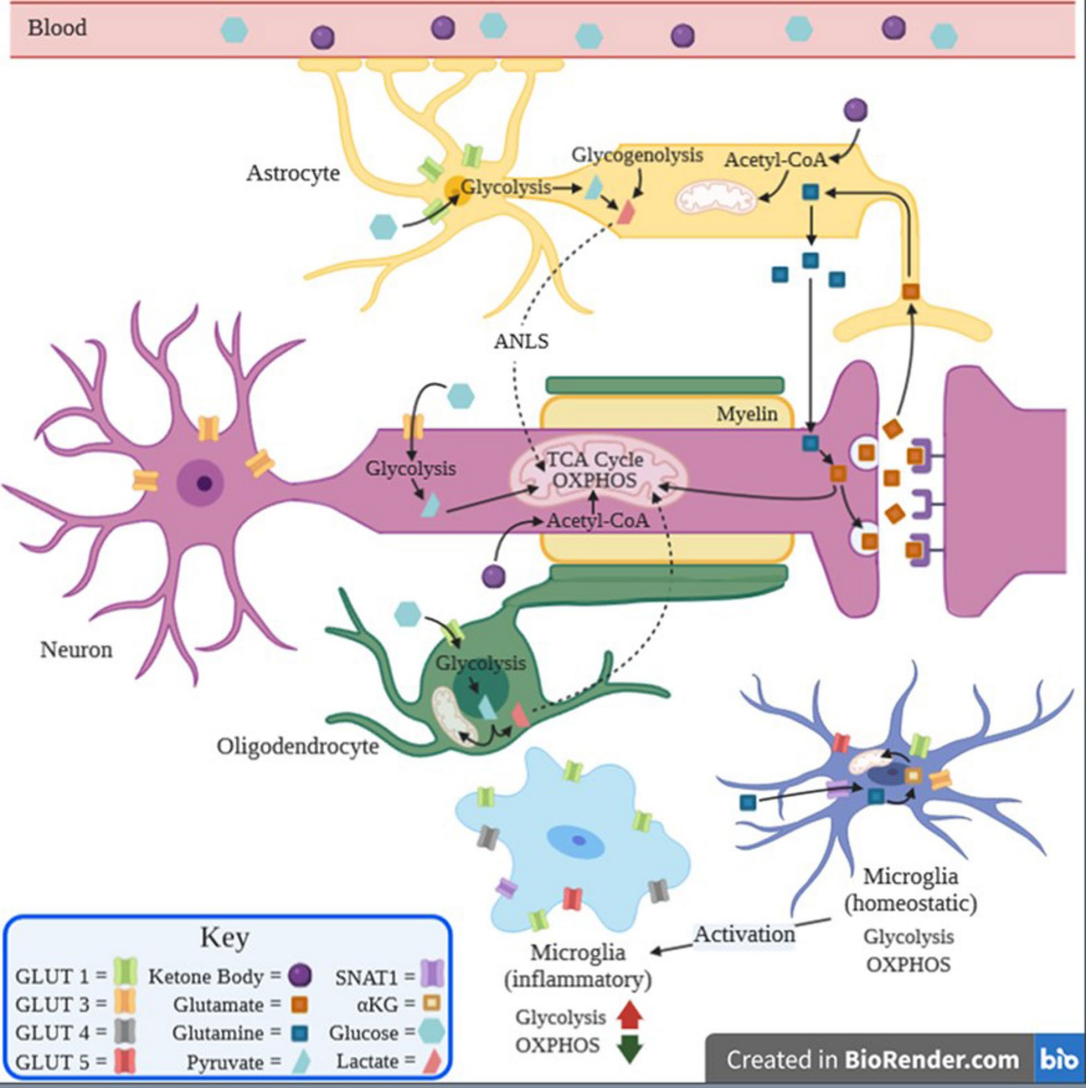
The metabolism of astrocytes (yellow), microglia (blue), neurons (pink), and oligodendrocytes (green) are intricately linked and change depending on substrate availability. The neuron relies primarily on glycolysis, the TCA cycle, and OXPHOS to meet metabolic needs. In times of low glucose, it receives lactate from astrocytes and oligodendrocytes to fuel its TCA cycle and generate reducing agents for OXPHOS via the ANLS. Neurons and astrocytes can also turn to ketone bodies produced by the liver and secreted into the blood in times of hypoglycemia. A key role of astrocytes is removal and conversion of glutamate from the synaptic cleft into glutamine which can be used in the TCA cycle by microglia and neurons

Neuronal activity, which involves action potentials and synaptic transmission, accounts for the majority of neuronal energy usage [3, 16]. At baseline, neurons are capable of generating ATP through complete oxidation of glucose to CO_2_ via glycolysis, but in a healthy state, were found to favor oxidative pathways, like the TCA cycle and OXPHOS [8, 17–19]. This stems from their inability to increase their glycolytic activity in response to cellular stress, which would lead to the generation and buildup of toxic glycation byproducts, ultimately causing apoptosis [8, 20, 21]. The substrate for neuronal OXPHOS is a source of controversy, with some reports pointing towards the existence of an astrocyte-neuronal lactate shuttle (ANLS) where astrocytes provide neurons with lactate to fuel the TCA cycle [8, 16, 17, 22–25] (see Fig. 1). Opponents of this theory base their opposition on theoretical and modeling studies that found neurons to have a larger capacity for glucose transport than astrocytes, supporting the view that glucose and not lactate is the primary substrate for neuronal oxidative metabolism [26–28]. Magistretti et al. rebuts these claims citing that glucose phosphorylation by hexokinase, not transport of glucose into the cell, is the rate-limiting step in glycolysis [16]. In addition, haploinsufficiency in neuron-specific glucose transporters (GLUT3) does not result in a pathological phenotype, whereas haploinsufficiency in astrocyte-specific glucose transporters (GLUT1) does [16, 29, 30]. Cultured neurons have also been found to prefer lactate over glucose when both are available [6].

Astrocyte end-feet cover much of the capillary surface and reside at every synapse, allowing them to both control the neuronal uptake of blood-borne nutrients and take in more glucose than other brain cells [31]. A key role of astrocytes in maintaining brain homeostasis is the removal of neurotransmitters from the synaptic cleft. The removal of neurotransmitters like glutamate is crucial for neurons as it can cause excitotoxicity. Glutamate is then converted to glutamine before being shuttled back to the neuron. This recycling and neuroprotective role of astrocytes is energetically expensive and requires a large amount of ATP [23, 32]. Like neurons, astrocytes can oxidize glucose to CO_2_; however, they have high glycolytic activity and low oxygen metabolism [7, 8, 32–34]. Increased glycolytic rates along with increased lactate export in astrocytes have been found to be markedly upregulated in response to glutamate stimulation. The lactate released into the extracellular environment can then be taken in by neurons and used in oxidative metabolism, as per the ANLS hypothesis. Astrocytes also possess a glyoxalase system that provides them with a pathway to detoxify the otherwise toxic byproducts of glycolysis [35]. With this, astrocytes are able to significantly upregulate and rely on glycolysis as a means of ATP production in a way that is inaccessible to neurons, while simultaneously providing substrates for the TCA cycle in neurons.

In hypoglycemia, astrocytes and neurons are forced to turn to alternative substrates. In the early stages of hypoglycemia in a healthy brain, astrocytes can utilize stored glycogen to produce lactate via glycogenolysis, which in turn can fuel neuronal metabolism [36]. This pathway is only available to astrocytes as they are the only location of glycogen storage in the brain, with total brain stores ranging from 3 to 12 μmol/g of tissue [37]. When these stores run out, astrocytes and neurons must then turn to ketone bodies (KBs), such as acetoacetate, acetone, and β-hydroxybutyrate. KBs are made in the adult liver in states of hypoglycemia and cross the blood–brain barrier (BBB), where they are converted to acetyl-CoA and used in the TCA in neurons and glial cells [7] (see Fig. 1). In various disorders including some neurodegenerative diseases like Lafora disease, there is aberrant glycogen metabolism that can lead to neuronal damage [38]. Recent studies have also found evidence of glycogen accumulation in the brain in glycogen storage disease type II, also known as Pompe disease [39].

Fatty acid (FA) metabolism is used for energetic and signaling purposes in glial cells and plays a signaling role in neurons. However, the contribution of fatty acids to substrate metabolism in neurons and glia is debatable and remains an emerging area of investigation. Recent research on neuronal fatty acid metabolism has focused on a brain-specific carnitine palmitoyltransferase 1 C (CPT1C). Unlike other CPT1 isoforms found predominantly in peripheral metabolic tissues, CPT1C is not involved in the β-oxidation of fatty acids and is not found in mitochondria [40]. Instead, CPT1C appears to play a regulatory role via its interaction with malonyl-coenzyme A (malonyl-CoA) [9, 41, 42]. Malonyl-CoA is the first product in the process of de novo fatty acid synthesis [9]. In hypothalamic neurons of the arcuate nucleus, the inhibition of malonyl-CoA by CPTC1C can regulate food intake [9].

In addition to the liver, astrocytes are also capable of making KBs from fatty acids [7, 43]. This process starts with β-oxidation of the fatty acid to make acetyl-CoA which can enter either the TCA cycle or ketogenesis. The role that KB production has in brain metabolism is not fully understood, but it has been hypothesized that it may provide metabolic support in states of starvation or ischemia and provide substrates for anaplerotic replenishment of neurotransmitters [7, 44].

### Oligodendrocytes

Oligodendrocytes (OL) are chiefly associated with the synthesis of lipid-rich myelin to form the myelin sheath that supports neuronal health and signal transmission. Oligodendrocytes have been demonstrated to use glycolysis and the TCA cycle to generate ATP to fuel biological processes, as well as the PPP for pyruvate carboxylation [45] (see Fig. 1). They have been also found to have the highest rate of oxidative metabolism of all brain cells [46]. It is likely that their metabolic requirements are tightly linked to the need for myelin synthesis. For example, recent studies using fatty acid synthase (FASN)-depleted OLs, have shown that endogenous FA synthesis is required for myelination during development and for efficient remyelination in adulthood [47]. Although it remains to be experimentally determined, this would suggest that increased NADPH recycling via the PPP and citrate production via the TCA cycle in OLs may be required to provide intermediates for FASN to drive myelin synthesis. While OL substrate requirements require further study, it is of note that provision of a high-fat diet can partially rescue myelin deficits OL-specific FASN KD mice [47].

### Microglia

Microglia, the brain resident macrophages have pleiotropic roles in the central nervous system (CNS). To perform a variety of functions, they express numerous cell surface receptors, including but not limited to toll-like receptors (TLRs), CD11b, and triggering receptor expressed by myeloid cells 2 (TREM2) [48–50]. The roles of these receptors in microglial function and how they could be harnessed for clinical diagnosis and treatment of CNS disorders continues to be a subject of intense research. However, it is becoming fairly well established that in response to invasion or injury microglia can phenotypically switch in response to specific environmental cues, and take on several changes including cell surface receptor expression, release of inflammatory cytokines, generation of reactive oxygen species (ROS), and changes in substrate utilization and metabolic requirements [48, 51, 52].

Transcriptomic studies suggest that microglia can use glucose, FAs, lipoproteins and glutamine as substrates for metabolism [51, 52]. Recent studies using CPT1 inhibitors to block mitochondrial fatty acid oxidation (FAO) in microglia showed increased microglial ramification and reduced motile surveillance and damage sensing capabilities, suggesting that FAO occurs in microglia and may drive function [53]. However, these studies are complicated by the fact that CPT1a is only expressed in astrocytes, implicating that mitochondrial β-oxidation is unlikely in microglia. It remains to be determined whether FAO could occur in microglial peroxisomes, but recent findings showing that inactivation of multifunctional protein-2 (MFP2), a pivotal enzyme in peroxisomal β-oxidation, leads to fatal neurological disorder characterized by microglial dysfunction suggest that peroxisomal β-oxidation may be an important and understudied facet of microglial metabolism [54]. Functional metabolic studies, particularly to determine whether FAs are utilized for fuel in microglia, either by mitochondrial or possibility peroxisomal β-oxidation, or rather intermediates for structural components or signaling molecules are much needed.

Under homeostatic, anti-inflammatory conditions microglia are believed to rely primarily on glucose and OXPHOS to meet their energy demands whereas pro-inflammatory ‘activated’ microglia have been found to rely more heavily on glycolysis [51, 52, 55]. In support, in homeostatic conditions microglia only express the glucose transporters GLUT3 and GLUT5. However, upon stimulation by lipopolysaccharide (LPS) and interferon gamma (IFNγ), microglia upregulate the expression of GLUT1 and GLUT4 as well as hexokinase 2 (HK2) a key glycolytic enzyme [51, 56, 57]. This shift in GLUT expression may represent the greater glycolytic need associated with pro-inflammatory activation and is reminiscent of the Warburg effect, which is often been reported in cancer cells [42, 58]. In light of recent findings suggesting that microglia in fact exist in a plethora of “activation” states, it is likely that changes to substrate utilization following “microglial activation” is more complex than previously thought. Since microglial activation plays a major role in the pathogenesis of all neurodegenerative diseases, and particularly AD, further studies aimed at defining the metabolic characteristics microglia are much needed. It is worth noting that recent transcriptomic studies that have highlighted the importance of distinct metabolic phenotypes in disease have used in vitro cell cultures or cells that have been isolated from the brain [12, 13, 59–61]*.* The ability of microglia to rapidly respond to their environment could affect the validity of these studies. For example, it has also been noted that microglia grown in cultures tend to appear more “activated” than microglia in their native environment [62]. This calls for the development of new methodologies that assess the metabolic profile of microglia removing them from their native environment.

## Metabolism in the brain during damage and disease

Neurodegenerative diseases affect millions of people worldwide and can be devastating for the afflicted and their families [63]. Of these diseases AD is the most common [2, 64], and will become more prevalent as the world’s population gets older [65]. Importantly AD, PD and MS share metabolic characteristics such as neuroinflammation and mitochondrial dysfunction. For example, during neuropathogenesis metabolic homeostasis becomes lost, which is associated with glial activation, neuronal damage, and cell death [66, 67]. Existing treatments for these diseases are not curative and at best slow the progression of the disease, highlighting the need to further understand metabolic aberrations underlying disease onset to develop new metabolism-focused intervention strategies with the potential to reverse or halt disease progression. Here, we will review our current understanding of the metabolic changes involved in AD, PD and MS.

### Alzheimer’s disease

AD is the most common cause of dementia. It was first described by Alois Alzheimer in 1907 who characterized it as a disease of progressive dementia with fibrils that were chemically distinct from the surrounding tissue [68, 69]. With the advent of advanced microscopy techniques the disease has been further characterized by formation of plaques in the brain consisting of amyloid beta (Aβ), neurofibrillary tangles (NFTs), and loss of neuronal synapses [70]. It is estimated that around 44 million people live with dementia worldwide, a number that is expected to triple by 2050 [71]. While many cases present sporadically, some genetic risk factors have been identified of which the Apoprotein E (APOE) ε4 allele presents the largest risk. Heterozygous APOE4 carriers have a reported odds ratio for AD of 3, whereas homozygous APOE4 carriers have an odds ratio of 12 [68, 72].

The APOE protein is a major component in glia-derived high-density lipoprotein (HDL)-like lipoproteins and is the primary apolipoprotein in CNS lipid metabolism. It is also involved in cholesterol and phospholipid efflux and clearance of lipids from the brain [73–75]. The APOE4 isoform has been found to be a significantly less potent acceptor of cholesterol and lipids than the APOE2 and APOE3 isoforms [73, 74, 76, 77]. This could be due to APOE4 lacking cysteine residues and leading to a propensity for posttranslational C-terminus cleavage preventing it from forming homodimers that allow it to accept lipids and to form lipoproteins [78–81]. This decreased lipid carrying capacity may lead to decreased lipid clearance from the brain, reduced lipid transport, reduced neuronal protection, and increased lipid accumulation, particular in microglia where lipid droplet accumulation may lead to cellular dysfunction [82]. Therefore, it is plausible to suggest APOE4-mediated defects in lipid and lipoprotein processing are central to the neuropathogenesis of AD. In support, astrocytes expressing the APOE4 isoform were found to accumulate more and smaller lipid droplets (LDs) than those with the APOE3 isoform [81]. Accumulation of LDs is indicative of cell dysfunction and a pro-inflammatory state, suggesting that cells expressing APOE4 isoform are more prone to activation and dysfunction that exacerbates the development of AD [81–84].

Recent studies have also found sex-dependent differences in APOE4 influences on metabolism. Female APOE4 carriers were found to have higher cerebral glucose metabolism than non-carriers while male APOE4 carriers displayed no significant difference [85]. This could be due to an interaction between estrogen receptors, estradiol, and APOE4 as suggested by literature, but clinical studies yield inconsistent results, and further studies are much needed [85–89].

Microglial activation and astrogliosis in response to amyloid deposition has been implicated in disease progression [70, 72, 90]. These phenotypic changes are coupled to metabolic alterations. Astrocytes and microglia have been found to switch to glycolysis in mouse models of AD, producing more lactate and exhibiting less TCA cycle activity [64]. This seems to contradict findings from fluorodeoxyglucose positron emission topography studies (FDG-PET) suggesting that the brains of AD and PD patients exhibit glucose hypometabolism [46, 91–93]. However, it is plausible that relative hypoglycemia may be representative of aberrations in systemic metabolism that are becoming increasingly associated with AD risk [94, 95]. It should also be noted that blood glucose levels also affect FDG-PET measurements, as the FDG competes with glucose to bind hexokinase [96]. In hypoglycemia, FDG will bind hexokinase more readily and not have as much competition due to relatively lower glucose levels. Similarly, in hyperglycemia, FDG will bind its target less readily as there is more relative competition. This confounds FDG-PET findings in neurodegenerative disease and should be considered during study design and data interpretation.

It has been proposed that inflammatory cells in the AD brain consume enough glucose to mask the metabolic deficits in neurons [97]. For example, metabolic tracer studies in a rodent model of AD have shown that AD neurons exhibit less TCA cycle activity and decreased expression of key mitochondrial enzymes [98], leading to poorly maintained ionic gradients and neuronal dysfunction. In addition, the loss of astrocytic TCA cycle activity cuts off neuronal supply of neurotransmitters, leading to an inability to maintain neurotransmitter homeostasis [98]. The interaction between neurons and astrocytes that both exhibit metabolic alterations in AD, leads to a cascade of events that culminates in neuronal death and cognitive decline, further supporting the need to understand this sequence of events in a temporal and cell-specific manner.

Fatty acid metabolism is associated with the pathogenesis of AD. Current research has focused on omega-3 and omega-6 poly unsaturated fatty acids (PUFAs) for the anti-inflammatory and pro-inflammatory effects of their breakdown products. The breakdown products of omega-6 PUFAs, like arachidonic acid (AA), promote inflammation and have been associated with increased AD pathology [99, 100]. On the other hand, omega-3 PUFAs are believed to have an anti-inflammatory effect [101]. However, this notion is often inconsistent with experimental findings. For example, increased levels of tissue omega-3 PUFAs have been associated with worse cognitive function in AD patients, suggesting that perturbation of fatty acid metabolism is implicated in AD pathogenesis [99]. Although it has been long postulated that supplementation with omega-3 PUFAs may be beneficial for patients with AD, clinical studies are also contradictory. While several studies have found that omega-3 PUFAs supplementation can improve cognitive function in both humans and animal models of AD [102–104], supplementation with the omega-3 PUFA docosahexaenoic acid (DHA) was not effective in preventing AD progression [105]. There are likely several reasons for these conflicting findings, including insufficient dosing regimens, the method of omega-3 delivery to the brain, and inherent defects in lipid processing that may simultaneously contribute to disease and unresponsiveness to omega-3 supplementation. Further research is needed to determine how changes in lipid metabolism may contribute or protect against the development of AD, particularly since dietary regimens that target lipid processing in the brain are a promising strategy to improve outcomes in patients with AD.

### Parkinson’s disease

PD is the second most common progressive neurodegenerative disorder in the United States. It is clinically characterized by a resting tremor, bradykinesia, stooping posture and rigidity. It is associated with cognitive impairment, neurobehavioral disorders like depression and anxiety, and autonomic dysfunction [106]. Pathologically, PD is characterized by dopaminergic neuronal loss in the substantia nigra and intracellular α-synuclein deposition and accumulation in cholinergic and monoaminergic brainstem neurons [106–108]. Unlike with AD, most cases of PD are sporadic although familial forms have allowed for the identification pathways involved in the disease. Some of these pathways include oxidative stress, axonal transport, calcium homeostasis, α-synuclein proteostasis, mitochondrial function, and neuroinflammation. Like AD, magnetic resonance imaging and PET studies have found glucose hypometabolism in PD patients [91, 93, 109]. Decreased levels of PPP enzymes have been identified in PD patients along with mutations in genes encoding α-synuclein, Parkin, PTEN Induced Kinase 1 (PINK1), Leucine Rich Repeat Kinase 2 (LRRK2), and DJ-1 in familial forms of PD [110, 111]. Decreased levels of PPP enzymes while not directly deleterious, impair the cells’ ability to make pentoses necessary for ATP generation and DNA repair. Mutations in the genes found in familial PD have been implicated in mitochondrial dysfunction, inhibiting OXPHOS and forcing cells to rely on glycolysis to generate ATP [110, 111]. While astrocytes are less dependent on OXPHOS, neurons rely heavily on mitochondrial respiration to meet energy demands. Therefore, impaired oxidative metabolism in neurons will disrupt neuronal functions and continue to the progression of PD.

Amyloid deposition is also seen in PD and has been hypothesized as a cause for microglial activation [93]. Several studies have shown that microglial activation is increased in PD patients and may exacerbate neurodegeneration in PD brains [64, 112]. Edison et al., used [(11)C](R)PK11195-PET to measure the upregulation of translocator protein, a marker associated with microglial activation in patients with PD and Parkinson’s disease dementia (PDD) [93]. They found that patients with PD showed a significant increase in microglial activation, and that this activation was even more pronounced in patients with Parkinson’s disease dementia (PDD), but that microglial activation was an early event in the neuropathogenesis of PD and was independent of amyloid pathology [93]. Moreover, they found concurrent reductions in glucose metabolism in PD patients, supporting the notion that metabolic permutation may drive disease pathology.

These activated microglia are believed to be key to pathogenesis of PD and generate oxidative species that damage neurons [64]. It has also been suggested that neuronal damage could be due to increased secretion of glutamate from activated microglia, since increased extracellular glutamate leads to impaired neuronal mitochondrial respiration and an inability to generate sufficient levels of ATP [113]. This cascade of events ultimately leads to cell death [114], and highlights the importance of metabolic cross-talk between different cells of the brain in the development of PD.

### Multiple sclerosis

MS is a chronic inflammatory and neurodegenerative disease in which immune cells cause demyelination and neuronal damage leading to cognitive decline. The disease has multiple clinical, radiologic, and histologic presentations. It is currently not known whether the inflammatory or neurodegenerative process is primary or secondary, and it is likely that pathogenesis is variable among patients, thus giving rise to different presentations [46, 115–118]. Although the disease progression is variable, most patients experience relapsing remitting multiple sclerosis (RRMS) characterized by periods of muscle weakness, blindness, double vision, trouble with sensation, and trouble with coordination, followed by total or partial recovery. In roughly 50% of patients, RRMS proceeds to secondary progressive multiple sclerosis (SPMS) where patients experience progressive deterioration. SPMS is typically diagnosed based on a history of progressive worsening of symptoms following a relapsing course of the disease. In some patients, the disease causes progressive damage from the onset with no periods of relapse. This form is termed primary progressive multiple sclerosis (PPMS). The speed of deterioration occurs at a similar rate in PPMS as it does in SPMS [119].

Four types of lesions have been characterized in MS. 20% of MS patients have immunopattern I lesions have T-cell inflammation, activated microglia, myelin-laden macrophages, and active demyelination suggesting that activated macrophages release toxic products that drive the demyelination process [120]. Approximately 50% of patients have immunopattern II lesions. These lesions are characterized by T-lymphocyte and macrophage infiltration, immunoglobulin deposition and complement activation on myelin. Immunopattern III lesions are characterized by oligodendrocyte apoptosis, microglial activation, T-cell inflammation and loss of myelin-associated glycoprotein, 2,3-cyclic nucleotide-3-phosphodiestarase [120]. This form describes about 29% of MS patients. The final form, immunopattern IV lesions are defined by non-apoptotic oligodendrocyte death and comprise only 1% of MS patients.

Regardless of the form, metabolic disturbances are central to the pathogenesis of MS. For example there is a growing body of evidence to suggest that mitochondrial dysfunction may underlie the neurodegeneration in MS, and is often present at the onset of clinical symptoms [121]. Dysfunctional mitochondria over-produce ROS, which can cause damage to proteins, lipids, DNA, and mitochondrial DNA (mtDNA) leading to cell damage and death [122–124]. OLs are particularly susceptible to damage by ROS due to their high metabolic rate and reliance on mitochondrial respiration to fuel the production of myelin proteins. Neurons are also susceptible to damage by ROS as they rely heavily on the TCA cycle to generate ATP. Astrocytes on the other hand are more glycolysis-dependent and therefore less affected by mitochondrial dysfunction and damage. Microglia become activated in response to the release of inflammatory mediators that are released by other microglia or infiltrating macrophages leading to further ROS generation and damage to nearby cells [125]. Microglia switch increased glucose utilization and increased fatty acid synthesis due to activation of mTOR and disruption of the TCA cycle [125]. Intracellular metabolites like malonyl-CoA in activated microglia also prevent fatty acids from associating with carnitine acyltransferase, preventing their entry into mitochondria, and thus inhibiting FAO [125].

Like AD and PD, metabolic alterations appear to be a critical component, if not causative, to the development and progression of MS. Importantly, AD, PD and MS are all characterized by the lack of a true disease-modifying therapy that is able to reverse or halt disease progression. This can be due to many factors like drug half-life, penetration of the active form of a drug into the BBB, the timing of the intervention, or ability of the drug to reach its target, inadequate bioavailability of the drug, or patient intolerance of therapy to name a few. Regardless of the reason for the lack of disease-modifying therapy, it is plausible to suggest that therapeutics aimed at restoring mitochondrial function may be an improved strategy that may rescue an increased susceptibility to develop ND in some individuals [126, 127]. To validate this hypothesis, further research is needed to define the time, region, and cell-specific changes in oxidative metabolism. Although current imaging modalities can measure changes in brain metabolism, the resolution is often lacking to determine cell-specific metabolic alterations. Going forward we will review the current range of methods used to visualize brain metabolism and propose FLIM as an emerging modality with sufficient resolution to empirically determine metabolic changes leading to ND.

## Metabolic imaging of the brain

It is clear by now that neurodegenerative diseases are accompanied by metabolic alterations in one or more cell types of the brain. Therefore, examining these alterations is a crucial step in understanding the etiology and progression of these diseases. There are many ways to study metabolism in cells and tissues such as mass spectrometry, activity and colorimetric assays, seahorse XF analyzers, and imaging [128]. Specifically, metabolic imaging is advantageous in a sense that it provides the spatial and temporal information that other techniques lack. Such spatiotemporal measurements are essential to resolve the metabolic changes in the different regions of the brain, as well as at different time points for longitudinal studies of disease progression.

Metabolic imaging modalities can be primarily divided into either ionizing radiation methods or non-ionizing radiation methods. Ionizing radiation methods include nuclear imaging techniques such as positron emission tomography (PET) and single-photon emission computed tomography (SPECT). Whereas non-ionizing radiation methods include magnetic resonance imaging (MRI) techniques such as magnetic resonance spectroscopy (MRS), hyperpolarized magnetic resonance, chemical exchange saturation transfer (CEST), optoacoustic (photoacoustic) imaging, as well as fluorescence microscopy imaging methods [129]. Coherent Raman scattering (CRS) microscopy [130] and mass spectrometry imaging (MSI) [131, 132] are other non-ionizing radiation methods which have been used to image metabolism in cells and tissues; although the latter does involve desorption/ionization of analytes by means of absorbing short ultraviolet (UV) wavelengths that are non-ionizing per se. To be clinically relevant, metabolic imaging techniques need to be noninvasive or minimally invasive (i.e., no biopsies and tissue sampling are required), and preferably without the use of exogenous tracers. Moreover, they need to provide enough resolution to differentiate between different cell types at least, if not at a subcellular level.

In vivo changes in brain metabolism have been assessed using the following noninvasive or minimally invasive neuroimaging techniques: MRI, functional magnetic resonance imaging (fMRI), MRS, diffusion weighted imaging (DWI), diffusion tensor imaging (DTI), PET and SPECT [133], in addition to functional photoacoustic microscopy (PAM) [134] (a focused review on advanced MRI and MRS methods used in brain metabolic imaging can be found in [135]). On the other hand, postmortem ex vivo examination of these changes has been generally performed using microscopy techniques [133] such as immunohistochemistry (IHC) [136], fluorescence microscopy with a variety of biosensors [137] (although such biosensors have also been used in in vivo brain metabolic imaging [138]), stimulated Raman scattering (SRS) [139]—which is a CRS technique, MSI techniques [140–142], and more recently fluorescence lifetime imaging microscopy (FLIM) [143–145] (though FLIM has also been used in in vivo brain metabolic imaging [53]). Autoradiography is another technique that has been used to observe changes in brain cell receptor expression or architecture [146–148]. Here, a radioactive tracer is injected into the sample prior to brain isolation and exposure of a radiation sensitive imager plate [149]. This technique provides good data on the regional localization of the target of a given radioactive tracer. Depending on the tracer, inferences can then be made on changes in metabolism. This technique has been applied to analyze changes in PD, AD, and TBI [146–149]. This technique is particularly useful to determine drug or ligand occupancy of specific-receptors and is therefore a gold-standard technique in pharmacological studies evaluating novel drugs, and their targets [150, 151]. However, the use of radiation, and the ex vivo nature of this approach is not always desirable.

Additionally, brain imaging methods can be broadly divided into three categories based on the information they provide: structural imaging, functional imaging, and molecular imaging. Structural imaging modalities include techniques such as computed tomography (CT) and MRI. CT images are created by exposing a patient to a rotating beam of X-ray radiation, whose signal is then collected and reconstructed to form a 3D image [152]. This can be used in conjunction with intravenously administered contrast agents, e.g., CT angiography, to allow for better imaging of vasculature [152, 153]. In the clinic, CT is useful for its quick acquisition time and is often used to rule out acute pathology such as hemorrhage, ischemia, or lesions. However, magnetic resonance images can provide better structural resolution especially when it comes to ND-associated structural changes. These images are created by exciting hydrogen ions and detecting energy release to construct an image [153]. Variations of MRI such as structural MRI and DTI have also been developed to study brain volume and tissue characteristics, and the integrity of white matter tracts [154].

Many MRI-based modalities have been developed to provide functional and molecular information. The most prevailing functional neuroimaging modality is blood oxygen level-dependent (BOLD) fMRI. This method relies on differences in the magnetic resonance signal created by changes in blood flow to brain regions, and differences in the magnetic properties of oxygen-rich and oxygen-poor blood. This provides an indirect measure of neural activity by associating metabolic activity with hemodynamic parameters that imply increased delivery or availability of nutrients to a specific brain region [155]. BOLD fMRI can be used for mapping brain networks associated with tasks or stimuli [156]. A similar technique is arterial spin labeling (ASL) wherein arterial blood is magnetically labeled and acts as a tracer to then measure differences in cerebral blood flow [157]. Rather than directly measure metabolism, these techniques provide good information on cerebral blood flow that can then be used to make inferences on regional cerebral metabolism.

PET is a well-established method of imaging that can provide metabolic information; the metabolic activity of brain regions can be imaged and analyzed using radioactive forms of glucose, dopamine, or radioligands for the 5-HT2 receptor, serotonin transporter, or D2 receptor [158, 159]. ^18^F-fluorodeoxyglucose (FDG) is one such tracer that has been developed to study regions of altered glucose metabolism in the brain. This is now widely used to study regions of infection, inflammation, or tumor growth [158, 160]. It should be noted, however, that localization of FDG is a measure of glucose uptake and does not provide a precise measure of glucose metabolism [96]. As previously mentioned, FDG will bind hexokinase but not be metabolized.

Each of the aforementioned methodologies has its merits. Nonetheless, there exist limitations to their usability and suitability for the metabolic research in question. PET technique, which is one of the most commonly used metabolic imaging methods for the brain- in addition to MRS—is limited by the high cost accompanying the production of its radiopharmaceuticals as well as the inability to distinguish anatomic details [161]. MRS limitations include potential tissue heating from the applied radio frequency (RF) energy, lengthy infusion and sampling schemes of the isotopically labeled substrates, and more significantly the metabolic composition retrieved from a given voxel may belong to different tissue types [161], and thereby depreciating the importance of brain tissue-dependent metabolite heterogeneity. Additionally, some MRS techniques (carbon spectroscopy) require either long acquisition time or infusion of ^13^C-labeled substrates, while others (hyperpolarization-based) are limited by their hyperpolarized metabolic probe’s short relaxation time [162]. Since many of these also rely on cerebral vasculature to either determine differences in brain regions or to transport a substrate, making these measurements susceptible to confounding by anatomical variation, as well as pathology that alters pericytes, endothelial cells, and smooth muscle cells which play a large role in controlling cerebral blood flow [163, 164]. Admittedly, many diseases also affect cerebral microvasculature, but this complicates separating metabolic differences from vascular differences [165].

The previously mentioned methods provide good structural, functional, and molecular information especially when used in conjunction with one another. However, they only provide regional resolution and are dependent on the detectable metabolites. On the other hand, optical imaging modalities provide cellular resolution. In light of these differences, an optical imaging modality such as FLIM, may be a better tool to study metabolic changes in the brain [15].

## Fluorescence lifetime imaging microscopy as a tool to measure in situ brain metabolism

Reduced nicotinamide adenine dinucleotide (NADH) and flavin adenine dinucleotide (FAD) are endogenous molecules in cells and tissues that have weak natural fluorescence [166]. Using specific wavelength of a light source, they can be transformed into an excited state with subsequent relaxation back to the ground state followed by simultaneous emission of a light signal of a different wavelength. The first NADH fluorescence imaging on live cells was performed in 1957 by Duysens and Amesz [167]. Since then, it has been widely used to study metabolic state of cells and tissues. NADH and FAD have divergent emission spectra with a little spectral overlap, which allows them to be imaged simultaneously. Fluorescence microscopy of NADH and FAD molecules helps to visualize where metabolic alterations are taking place in biological samples, including cells and tissue sections.

While the fluorescence intensity signal of NADH and FAD is highly informative and represents the activity of metabolic pathways, it is proportional to their concentration in the imaged samples. This can become problematic when reproducing measurements or when a fluorophore is only present at small concentrations in a sample. One way to circumvent this reliance on fluorophore concentration is to look at fluorescence lifetime instead of fluorescence intensity. With fluorescence lifetime the amount of time a fluorophore spends in the excited state is measured. This property of a fluorophore is dependent only on the molecule’s microenvironment and not on its concentration [168–170]. This can be particularly advantageous in the field of biology as some traditional fluorophores are cytotoxic or not native to a cell’s environment. As previously mentioned, FLIM allows for the use of endogenous fluorophores that are already present in biological systems [167, 168, 171]. Looking at a fluorophore’s fluorescence lifetime as opposed to its fluorescence signal intensity also eliminates the requirement that a fluorophore displays spectral shift in response to a change in environment [168]. This also allows for the use of fluorophores with large wavelength absorption spectra, potentially lowering the cost of instrumentation [168].

Development of laser scanning microscopy methods in conjunction with fluorescent molecules lifetime measurement marked the advent of FLIM. With FLIM, contrast in an image is generated by differences in fluorescence lifetimes [168]. This new method allows for the study of a fluorophore’s microenvironment, shedding light on the different environments in a cell or tissue. Examples of factors that can change a fluorophore’s fluorescence lifetime include the presence of oxygen, calcium, pH, energy transfer, as well as many other factors like nearby proteins or quenchers [172–184]. As the field grows, more fluorophores will enter the field. NADH and FAD, which have intrinsic fluorescence and their lifetime is tied to the state of the molecule [185, 186]. Given the subcellular resolution fluorescence microcopy provides, examination of these fluorophores allows for a direct analysis of the metabolic profile of individual cells.

Advances in the field of microscopy over the last century have been key in the development of FLIM. Two-photon excitation microscopy allowed for deeper imaging of tissues and reduced photodamage even with longer imaging times and allowed for the interrogation of femtoliter volumes in a sample [185, 187–191]. The ability to capture spatial and temporal resolution simultaneously in microscopy was also key in the development of FLIM as this allowed for fluorescence lifetime data acquisition in volumes small enough to analyze individual subcellular compartments in real time [189, 191].

The fluorescence lifetime of NADH changes depending on whether it is free or in a bound-to-enzyme form [186, 192]. For free NADH, the lifetime is around 0.4 ns versus around 3.4 ns when it is in a bound-to-enzyme form [15]. Other factors that affect the fluorescence lifetime include pH, temperature, and redox state. Since NADH is intricately involved as a cofactor in multiple metabolic pathways, differences in its lifetime can be used to determine which pathways a cell is using to meet its metabolic demands. FAD is another cofactor whose autofluorescence can and has been used to investigate metabolic processes in situ [193–196]. Specifically, FLIM has found a niche in analyzing tissue for markers of cancer, since a hallmark of cancerous cells is the reprogramming of metabolic pathways [197–203]. It should be noted that there are many other molecules in cells and tissues that exhibit autofluorescence and whose fluorescence spectra overlap with these probes, and techniques have been developed to bypass this problem [186, 192, 204, 205].

**Fig. 2:**
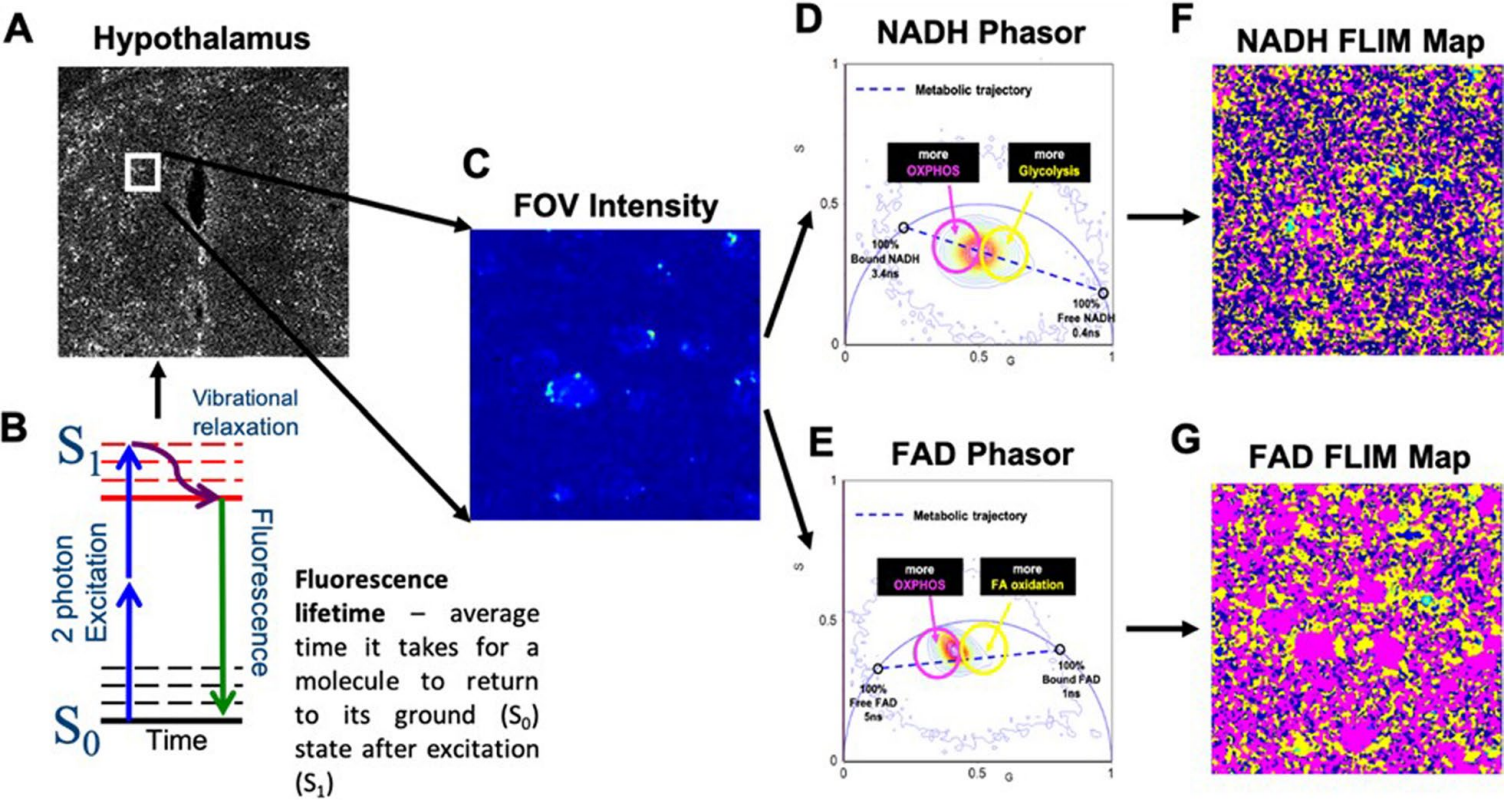
FLIM imaging and analysis. Mouse hypothalamus (**A**) is excited (**B**) with 2 photon laser to collect NADH and FAD fluorescence signal on multiple smaller fields of view (**C**) and measure their lifetimes. Phasor plots for NADH (**D**) and FAD (**E**) and corresponding lifetime maps distributions (**F** and **G**, respectively) are created

The phasor approach for FLIM data analysis helps to avoid multiexponential analysis used in typical fluorescence decay fitting [187, 189]. In phasor analysis each pixel of an image has its fluorescence decay plotted as a vector on a universal semicircle plot with vertices at (0,0) and (0,1). These points correspond to an infinite lifetime and a lifetime of zero, respectively. Pixels with contribution of multiple different lifetimes lie inside the circle while pixels with more homogenous lifetime composition lie closer to the periphery of the circle [187, 189]. The resultant phasor plot provides insight on the state of various fluorophores within a sample, which is not intended to determine exact fluorescence lifetimes (Fig. 2). Notably, there are caveats to this fit free analysis of FLIM data. Phasor analysis is not sensitive to low photon counts [206], and cannot reliably distinguish small changes in the redox state in the sample [186]. Moreover, the metabolic analysis does not have a single pathway resolution and response is reported by the ratio of free-to-bound fractions, where both numerator and denominator can change, while maintaining similar ratio [207]. Further, if the calibration decay is not acquired properly, this can lead to improper conversions of data to phasors [208]. Additionally, if the phasor transformation is not done with data acquired in entire period of the laser repetition rate, advantages of phasor plot analysis can be diminished [209]. The consequences of such errors as well as ways to avoid them are outlined elsewhere [185, 209].

As previously discussed, many neurodegenerative diseases involve metabolic reprogramming. By using FLIM, these changes in brain metabolism can be characterized with the possibility of identifying metabolic derangements prior to the onset of symptoms. For example, we have recently shown that neurons lacking the lipid processing enzyme lipoprotein lipase (LPL) show a shift in the NADH phasor plot indicative (Fig. 2) of increased free NADH compared to wild-type neurons, suggesting that LPL depletion results in metabolic shifts towards glucose utilization and away from oxidative metabolism (Fig. 2) [144]. These findings were the first to combine electrophysiological measurements of neuronal function with metabolic changes and highlight how FLIM may be used to measure metabolism alterations in fresh-frozen brain sections [144]. Recent studies have also employed FLIM to successfully determine metabolic changes in primary cultures of neurons and astrocytes in response to toxins that mimic the effects of PD [210]. Specifically, manganese (Mn) treatment leads to increased bound NAD(P)H in neurons consistent with enhanced apoptosis, but leads to decreased bound NADH in astrocytes, possibly due to a shift towards glycolytic metabolism and impaired respiration [210]. FLIM has also been used to study chronic neuroinflammation in the brains of mouse models of MS and in venous blood of human subjects [211–213]. Here, NAD(P)H was used as a fluorophore to detect active NADPH oxidase and as an analog for oxidative stress [211–213]. NAD(P)H can be used as an analog for oxidative stress because it serves as a source of electrons to generate ROS via NADPH oxidase [214–216]. In the brain, overactivation of NADPH oxidase has been linked to neurodegenerative disease [214–216]. This technique has also been applied to studying the pathophysiology of cerebral amyloid angiopathy and glioblastoma [217, 218].

With enough characterization of a given fluorophore, and its possible intracellular bound states and respective fluorescence lifetimes, one can generate a database that allows for near complete analysis of a cell’s metabolic state in various compartments [219]. Leben et al. undertook such an endeavor, generating a database of the various fluorescence lifetimes that NAD(P)H has when bound to various enzymes. With the help of RNA-seq data, they identified metabolic enzymes like malate dehydrogenase or lactate dehydrogenase, inducible enzymes like nitric oxide synthase and NAD(P)H oxidases, as well as other abundant small enzymes like sorbitol dehydrogenase that were most likely to bind to NAD(P)H and change its fluorescence lifetime [219]. By performing measurements of homogenous mixtures of NAD(P)H and single enzymes they were able to generate a database listing nearly all possible fluorescence lifetimes for this fluorophore [219]. This demonstrates that with enough fluorophore characterization, FLIM has the power to identify the state that a given fluorophore is in, what enzymes it is bound to, as well as what its microenvironment looks like in various intracellular or extracellular compartments. Although FLIM characterization is somewhat new, and often require validation studies to support the findings, it is clear even from the limited number of published studies that FLIM analysis can resolve cell-specific changes in brain metabolism that would otherwise be missed by alternative imaging modalities.

Microglia are of particular interest since altered microglial metabolism coincides with changes to phenotype and function that may contribute to, or protect against, the pathogenesis of many neurodegenerative diseases. However, existing methods for studying them rely on removing microglia from their native environment to study their gene expression and phenotype.

Recently, several publications have begun to utilize FLIM to understand inherent characteristics of microglial metabolism that may differentiate them from other cells in the brain. Sagar et al., measured the lifetime of NADH in both primary microglial cultures and in 100-μm-thick coronal sections from mice that had been treated with increasing doses of LPS in order to modulate the inflammatory status of the brain and “activate” the microglia [145]. Interestingly, they found that microglia in their activated state surveillant or inactivated state had a shorter NADH mean lifetime compared to other glial cells, and that they had a greater fractional contribution from free NADH [145]. In contrast, LPS treatment increased their mean NADH lifetime and decreased the fractional contribution of free NADH (compared to vehicle treatment) [145]. In support, Bernier et al., 2020 recently reported that resting microglia in situ have a much shorter NAD(P)H lifetime than surrounding non-microglial cells [53], and therefore a more glycolytic state. Importantly this study performed glycolytic inhibition with iodoacetate to confirm that glycolytic activity correlates with measurements of free short lifetime NADH [53]. Together, these studies suggest that microglia are much more glycolytic than previously thought, particularly in comparison to non-microglial cells and that activated microglia in fact may metabolically shift away towards oxidative metabolism to support the greater bioenergetic needs of activation-associated functions such as phagocytosis. Since these findings are somewhat in contrast to previous literature, and in particular findings from (albeit peripheral) inflammatory and macrophage models, it is imperative to further validate these findings using models of neurodegenerative disease such as AD, PD and MS. These discrepancies also highlight the importance of further work to validate our understanding of cell-and-disease-state specific brain metabolism, and to determine whether our findings corroborate with those from existing metabolic imaging approaches. In other words, will our findings using FLIM challenge our previous notions? For example, FDG-PET, described above, is predominantly used to measure brain glucose metabolism, which is interpreted as neuronal metabolism and neuronal activity. Since we know that non-neuronal cells majorly contribute to glucose uptake and metabolism, could glial cells also contribute to FDG-PET readouts? Although further method development is required, FLIM analysis of brain tissue from various metabolic and disease state paradigms, in conjunction with cell-specific markers, may eventually help answer these questions. Nonetheless, it is clear from the few studies employing FLIM, that this technique will enable major advancements in our understanding of microglial metabolism in situ. With more widespread use of FLIM and better metabolic characterization of microglia, FLIM could be used to identify mechanisms that promote or protect against the development of ND.

## Conclusion

A striking similarity between ND is the immunometabolic switching of glial cells. Specifically, in AD, PD and MS oxidative-to-glycolytic, and shifts are often observed. It is plausible to suggest that targeting glycolytic shifts may be a potential therapeutic strategy for the treatment of ND. This is particularly promising in the case of AD, since disease-modifying treatments are mostly lacking. Here we suggest that imaging modalities such as FLIM, that can discriminate between subcellular, cell-type and regional differences in substrate utilization may be instrumental in the development of strategies that target metabolic processes to modify cellular function and improve outcomes in patients with AD and beyond.

## Data Availability

Not applicable.

## Abbreviations

ND: Neurodegenerative diseases
AD: Alzheimer’s disease
PD: Parkinson’s disease
MS: Multiple sclerosis
FLIM: Fluorescence lifetime imaging microscopy
MRI: Magnetic resonance imaging
MRS: Magnetic resonance spectroscopy
PET: Positron emission tomography
NADH: Nicotinamide adenine dinucleotide
FAD: Flavin adenine dinucleotide
TCA cycle: Tricarboxylic acid cycle
OXPHOS: Oxidative phosphorylation
ATP: Adenine triphosphate
Acetyl-CoA: Acetyl coenzyme A
CO_2_: Carbon dioxide
O_2_: Oxygen
PPP: Pentose phosphate pathway
NADP^+^: Nicotinamide adenine dinucleotide phosphate
ANLS: Astrocyte-neuronal lactate shuttle
GLUT3: Glucose transporter 3
KBs: Ketone bodies
FA: Fatty acid
CPT1C: Carnitine palmitoyltransferase 1 C
malonyl-CoA: Malonyl-coenzyme A
OL: Oligodendrocytes
FASN: Fatty acid synthase
CNS: Central nervous system
TLRs: Toll-like receptors
TREM2: Triggering receptor expressed by myeloid cells 2
FAO: Fatty acid oxidation
LPS: Lipopolysaccharide
HK2: Hexokinase 2
Aβ: Amyloid beta
NFTs: Neurofibrillary tangles
APOE: Apoprotein E
HDL: High-density lipoprotein
PUFAs: Unsaturated fatty acids
PINK1: PTEN induced kinase 1
LRRK2: Leucine rich repeat kinase 2
RRMS: Remitting multiple sclerosis
SPMS: Progressive multiple sclerosis
mtDNA: Mitochondrial DNA
CEST: Chemical exchange saturation transfer
CRS: Coherent Raman scattering
UV: Ultraviolet
fMRI: Functional magnetic resonance imaging
PAM: Photoacoustic microscopy
IHC: Immunohistochemistry
CT: Computed tomography
BOLD: Blood oxygen level-dependent
FDG: ^18^F-fluorodeoxyglucose
